# Toward normalising abortion: findings from a qualitative secondary analysis study

**DOI:** 10.1080/13691058.2019.1679395

**Published:** 2020-01-14

**Authors:** Carrie Purcell, Karen Maxwell, Fiona Bloomer, Sam Rowlands, Lesley Hoggart

**Affiliations:** a MRC/CSO Social and Public Health Sciences Unit, University of Glasgow, Glasgow, UK; b School of Applied Social and Policy Sciences, Ulster University, Newtownabbey, UK; c Centre of Postgraduate Medical Research and Education, Bournemouth University, Bournemouth, UK; d School of Health, Wellbeing and Social Care, The Open University, Milton Keynes, UK

**Keywords:** Abortion, abortion stigma, normalising abortion, qualitative secondary analysis (QSA), UK

## Abstract

In most settings worldwide, abortion continues to be highly stigmatised. Whilst a considerable body of literature has addressed abortion stigma, what is less commonly examined are the ways in which those with experience of abortion describe it in non-negative terms which may resist or reject stigma. Drawing on qualitative secondary analysis of five UK datasets using a narrative inquiry approach, we explore: the use of non-negative language around abortion, potential components of a normalising narrative, and constraints on non-negativity. As such, we present the first empirical UK study to critically examine how a dominant negative abortion narrative might be disrupted.

## Introduction

In most settings worldwide, abortion continues to be commonly framed as controversial and highly stigmatised. A now considerable, predominantly US-focused literature has addressed this stigma, attributing it largely to the challenge abortion poses to powerful norms of feminine sexuality, underpinned by intersecting health inequalities (Kumar, Hessini, and Mitchell 2009; Norris et al. 2011; Cockrill and Nack 2013; Hanschmidt et al. 2016). This situates abortion stigma as the significant issue for equitable access to sexual and reproductive healthcare, and for the wellbeing of women undergoing abortion. What is as yet under-explored from a research perspective, and what we address in this paper, is what a shift in focus from stigma to normalisation might look like when grounded in women’s lived experience of abortion.

In the UK, a shift toward normalising abortion is evident in a nationwide, multiorganisation campaign for full decriminalisation of abortion.^1^ As with many current grassroots and research-based projects (such as My Body, My Life http://mybody-mylife.org), the decriminalisation campaign argues that normalising abortion as part of routine healthcare is essential to countering stigma and inequity (Dyer 2017). This time of heightened interest presents a significant moment to examine ways in which dominant, stigmatising narratives of abortion might be disrupted, and normalising narratives given greater credence.

Nevertheless, at a societal level, the prevailing default position on abortion tends to be that it is inherently bad. This in turn contributes to the perpetuation of ‘abortion negativity’ (Lee 2004), or the ‘awfulisation’ of abortion (Hadley 1997). An influential paper by Kumar, Hessini, and Mitchell (2009) on abortion stigma has been met with a flurry of research examining ways in which stigma negatively shapes the experiences of women undergoing abortion, those providing it, and in the wider community (e.g., Purcell, Cameron et al 2017; Harris et al. 2013). The relationship between stigma and negative cultural attitudes to abortion should not be considered causal, however. Rather, this is a dynamic circular relationship in which each influences the other, meaning negative cultural attitudes both produce, and are a consequence of, stigma. Moreover, there are other important aspects to this conversation, including fundamental inequalities which underpin and generate stigma (Kumar 2013); ways in which identities are (re)negotiated dynamically through language (Beynon-Jones 2017); and, we argue, action which might more actively work toward the normalisation of abortion.

Baird and Millar’s (2019) analysis of representations of abortion in popular culture and elsewhere considered how abortion narratives at the public discourse level may or may not contribute to normalising abortion. The authors identify the trope of the ‘unapologetic’ abortion narrative and suggest that, while this may contribute to ‘increas[ing] the cultural legitimacy of abortion’, it does not fully escape the dominant negative narrative of stigma and awfulisation (Baird and Millar 2019, 9). The research we present here speaks directly to their call for more scholarship to address the normalisation of abortion.

Research suggests that women’s responses to seeking and undergoing abortion may include negativity, positivity and ambivalence – that is, experiencing multiple emotions simultaneously – and that the complexity of feelings experienced by women warrants further attention (Kero 2014; Kero and Lalos 2000). What women who have undergone abortion feel able to say about their experiences, however, is constrained by the social narratives they perceive to be readily available to them (Beynon-Jones 2017; Macleod, Sigcau, and Luwaca 2011; Purcell, Brown et al. 2017). Popular culture and the media contribute to perpetuating particular narratives (Purcell, Hilton, and McDaid 2014; Sisson and Kimport 2016, 2017), although a multiplicity of experiences has become more evident in recent years (Sisson 2019). Women are also significantly constrained by powerful cultural (including religious and patriarchal) norms, which in turn lead to a silencing of discussion about abortion within society (Bloomer, O’Dowd, and Macleod 2017). Resistance to such norms has been identified, although the extent and roots of this resistance remain unclear (Bloomer, O’Dowd, and Macleod 2017; Hoggart 2017).

This paper draws on one component of a qualitative secondary analysis study – the Sexuality and Abortion Stigma Study (SASS) – which, in full, brought together 11 UK datasets relating to abortion. The original focus of the study was to explore manifestations of abortion stigma in the UK. As we began to collate and review the data, however, it became apparent that such manifestations were pervasive, and that we would need a refined focus, and multiple approaches, to tackle our exploratory analysis productively. It also became apparent that non-negative presentations of abortion were comparatively less common, but that exploration of these could be illuminating with regard to both stigma and normalisation. As one avenue of exploration, we therefore opted to turn the manifestations of stigma question on its head, and focused on exploring *absences* of stigma, instances in which participants potentially challenged or rejected stigma. We frame this as ‘non-negativity’ rather than ‘positivity’ to acknowledge that the absence of negativity did not always equate to explicit positivity. A qualitative secondary analysis approach offered the potential to interrogate across datasets what non-negativity might look like in multiple contexts, and what this in turn might contribute to debates around normalising abortion.

In taking this approach, we do not aim to privilege non-negative and positive attitudes to abortion to the exclusion of all others. Indeed, a key strength of exploring multiple datasets is the potential to represent an array of attitudes and experiences. We fully acknowledge the complex mix of feelings women often have about undergoing abortion. In highlighting non-negativity, we propose that drawing out these framings might contribute to a disruption of the default conceptualisation of abortion as negative and controversial, and thus offer an alternative basis from which to build a normalising narrative.

## Methodology

Qualitative secondary analysis is increasingly recognised as an effective means of adding value to original research by re-analysing data to bring new substantive and methodological insights, maximise learning from existing data, and inform health policy (Bishop and Kuula-Luumi 2017; Davidson et al. 2018; Tarrant 2017). This approach can be especially valuable in relation to ‘sensitive’ subjects or ‘hard-to-reach’ populations, where data production is challenging (Long-Sutehall, Sque, and Addington-Hall 2011; Tarrant 2017). Qualitative secondary analysis can combine the breadth of quantitative scope with the depth of qualitative insight (Bishop and Kuula-Luumi 2017; Davidson et al. 2018). In the context of the current study, it offered the opportunity to pool data from multiple studies sharing the same broad topic, whilst retaining the attention to detail and depth of analysis that characterise good qualitative research. Rigorous qualitative secondary analysis can arguably go further than other approaches that synthesise multiple studies, in that data are re-analysed, offering opportunities to pose new questions (Davidson et al. 2018).

Qualitative secondary analysis can be methodologically challenging (Davidson et al. 2018; Tarrant 2017): combining data collected at different times, with different aims and diverse populations, poses inherent difficulties. However, concerns that secondary analysts may be blind to contextual factors and concerns of the primary researchers and participants (Coltart, Henwood, and Shirani 2013; Davidson et al. 2018) can be ameliorated by close liaison with the primary researchers. In this study, all primary researchers were either co-investigators or members of the study advisory group.

For this paper, five datasets, comprising one-to-one interviews with 138 women who had undergone abortion were used. The original studies were conducted between 2008 and 2016 in Scotland and England (see Table 1 for details). These datasets have already generated a substantial body of literature (see Hoggart 2012, 2017, 2019; Hoggart, Newton, and Bury 2015, 2017; Purcell, Brown et al. 2017; Purcell, Cameron et al. 2014, 2016, 2017; Purcell, Riddell et al. 2017).

Our approach takes a phenomenological-sociological perspective, situating common ‘typifications’ – the background assumptions which facilitate social interaction, and which are shared through language – as constituting the building blocks of the social world (Schutz 1967). From this perspective, typifications accumulate over time, based on an individual’s experiences and what they perceive of those around them. This includes common high-level narrative tropes and stereotypes, such as the typifications of abortion as ‘bad’, and women who have abortions as ‘irresponsible’. These typifications in turn comprise a stock of knowledge, or scheme of reference, that is used in everyday life to interpret and shape accounts of lived experience.

Since this stock of knowledge is constituted through language, close attention to, and unpacking of, this language is essential to understanding it. As the language people use is drawn from the options they perceive to be available to them, it is culturally constrained and not of an individual’s making or choosing (Archer 2007). Moreover, much of what they say will be shaped by ‘real and imagined dialogue with what others think, do and feel’ (Holmes 2010, 148). We also therefore draw on a narrative inquiry methodology as a means of addressing the data, focusing on the stocks of knowledge which research participants constitute through talk, and what we can learn from these as ‘explanatory schema’ (Riessman 1990, 2008).

A flexible and systematic approach to analysis was required to ensure effective ways of analysing within and across datasets. We first reviewed the data to identify cases which could illuminate how abortion is framed when talked about in non-negative ways. Four accounts from different projects were identified as including non-negative talk, which were subject to detailed in-depth analysis for language choices and tropes drawn upon. This preliminary analysis highlighted common themes including: absence of regret; certainty about the decision; and resistance to internalising feelings of self-blame, shame or irresponsibility. Regarding types of language used, talk about the abortion in practical terms was common, as were framings such as being ‘at peace’ with the decision or and it being ‘the best decision for me’. Social support from significant others (partner, mother, friend) was another commonality, alongside a degree of ease in talking to others about their experience. These indicative findings were developed into a broad, flexible coding framework comprising potential components of a non-negative stock of knowledge on abortion, which was used in the next stage of analysis.

This approach also highlighted that all cases tended to contain a complex mix of negative and non-negative language, suggesting that a sole focus on one or the other would be limiting. We thus opted for a strategy of ‘amplified sampling’ (Heaton 2004), selecting transcripts at regular intervals from each dataset, which were ordered chronologically (e.g. every third or fourth transcript, depending on the size of the dataset). This generated an indicative snapshot across the dataset as a whole, which facilitated understanding how the types of language initially identified were articulated across women’s accounts.

The resulting sub-sample of 25 interviews was subjected to in-depth analysis. This stage involved repeated re-readings of the transcripts, followed by broad coding of relevant sections as per the framework. Coding was conducted by KM in close consultation with CP, with both meeting frequently to discuss findings, and further refine the analysis. Both also met regularly with LH, FB and SR, to discuss and develop interpretations and explore potential alternative explanations. Ethical approval was gained from the original institutions’ Ethics Review Committees, on the basis that participants in the studies had given specific consent for their data to be used in future research.

## Findings

Our analysis foregrounded several interrelated and overlapping features in the language used by women in accounts of their abortion experiences. These have in turn enabled us to address the question of what a stock of knowledge – what we refer to here for brevity as a ‘narrative’ – in which abortion is normalised might consist of; and what narrative resources (i.e. options) appear to be available to women who wish to account for their experiences in non-negative terms.

We first present a general exploration of non-negative framings, before examining in greater detail two potential components of a normalising narrative – explicit positivity and the use of negation statements – before addressing a notable set of constraints on non-negativity, which include ‘real or imagined dialogue with others’ about abortion. While we would usually favour the use of pseudonyms when presenting verbatim quotes, as is typical in qualitative research, in this instance the large body of data from different projects, and the anonymised format in which we received the shared data, increases the risk of participants’ actual names being inadvertently used, leading us to instead use transcript codes when presenting data extracts from this study. Codes denote the order in which transcripts were analysed in the QSA, and are followed by the woman’s age and geographical location.

### ‘I’ve done the right thing’: non-negative framings across the dataset

Examples of non-negative language and framings were evident across the data. These related to women’s reflections on the experience, the decision-making process, and were primarily articulated as reactions to how they might have been expected to feel.

SASS062’s reflections indicated some surprise that the experience was not as bad as she might have expected:

…the whole experience was actually OK, I wasn’t like traumatised by it and I wasn’t… I was quite practical about it. And then they [abortion providers] made me feel ‘yeah, if that’s what you think is right for you then that’s fine, we’re not going to, you know, be like “no, you’re doing wrong”’. So, you know, they assured me that it’s my decision and if that’s what I want to do then that’s fine. (SASS062, 21, Scotland)

As well as arguably speaking to a dominant narrative of abortion as difficult or ‘traumatic’, and how this contrasted with her actual experience, this woman’s explanation highlights the part played by providers in framing the abortion as her decision, and an acceptable one to make, in a way which she found to be beneficial.

Another woman, name, spoke to the decision-making process, noting:

[We were] probably quite mature about it. Well, I like to think I was quite mature about it. Obviously, it’s not an easy decision to make but I do think we made the right one… (SASS059, 21, Scotland)

For (name), the feeling of having acted ‘maturely’ can be read as indicating a sense of empowerment achieved through her experience. Her language choices also speak to a broad assumption that choosing abortion is ‘not an easy decision’ but that, regardless of this, it is nonetheless the right one for some people in some circumstances.

Abortion as having been ‘the right thing’ was a common non-negative refrain across the data. For example, another participant said: ‘I definitely, definitely made the right decision, because I feel like, you know, I’ve done the right thing’ (SASS124, 24, England).

As well as maturity and certainty, another factor women presented in non-negative terms was dealing with the situation ‘practically’ and without unnecessary ‘drama’:

I’m not one to make a fuss. I immediately knew exactly what I was going to do and that was the situation, so I have absolutely no qualms at all, like, I have no need to gossip, I have no need to cause drama, you know. Shit happens, you deal with it, you move on. It’s life. (SASS053, 23, Scotland)

This woman’s approach to dealing with the abortion was framed in a pragmatic way, with a clear sense of drawing a line under it once it was over.

One thing that is perhaps striking here is the absence of any sustained non-negative narrative of abortion as a woman’s right. As we show below, this did emerge in one or two instances of more explicitly positive talk. However, framings of abortion vis-à-vis women’s right to bodily autonomy, reproductive choice, reproductive/social justice, or any other aspect of the fundamental feminist argument for abortion, were primarily notable by their absence.

### Abortion as ‘an amazing option’: explicit positivity

The first component of a normalising narrative that we address in detail is the framing of abortion as a positive option. Explicit statements of positive feelings and attitudes were relatively rare – appearing in only a handful of accounts – but were nonetheless significant for the counterfoil they offered to dominant negative framings. A key example of the use of overtly positive language came from SASS033 and was evident throughout her interview. She initially explained:

I was so happy, you know, so happy. Because I was kind of prepared for the worst, that they will tell me that for no reason I have to wait one week or something. I just really didn’t want to be pregnant, even one day more so… (SASS033, 31, Scotland)

This ‘happiness’ permeated SASS033’s account and seems to have stemmed at least in part from the relief engendered by what she viewed as swift and supportive treatment from health professionals, compounded by the fact that she originated from a country where access is significantly more limited (to which she alluded elsewhere). She went on to explain that she did not feel abortion should be silenced, and that she was happy to discuss her experience:

I freely share my experience with whoever wants to listen. I mean, for me it’s not taboo, I speak with my friends about it, with my friends who are male also. I just tell them: ‘you know what happened? I was pregnant.’ I feel free to speak about it because, you know, it’s happened to other people. And the leaflet I got in the hospital says that at some point one third of women in the UK will have a termination of pregnancy. So plenty of women went through this. (SASS033, 31, Scotland)

Again, this excerpt suggests this participant’s own attitude to abortion interacted with the knowledge provided at the clinic, that she was one of a third of women in the UK who undergo abortion.

SASS033 went further to describe feeling ‘awesome’ immediately following the abortion, and that it could be an explicitly positive life event:

I think it really can be a positive experience, it doesn’t have to be anything dramatic, like for me it was very positive. Because the first time I smiled after a long time, it was on Monday when they told me I’m going to have the pill the same day, that’s when I smiled, and then I realised ‘wow, I was not smiling really for a long time’. (SASS033, 31, Scotland)

Her presentation of this point highlights her perception that abortion is often viewed as, in a negative sense, ‘dramatic’. On the whole, SASS033 explained that she views her experience as ‘a happy story because I feel very good now, you know, it [has] a happy end’.

Elsewhere in the data, more positive language about abortion related to the choice to seek it, and how that process was experienced. One participant explained:

Participant: I think my opinion is that it’s basically an amazing option that women have now. I don’t think it’s to be taken lightly, and it’s definitely not, you know, like a… It’s not an easy thing to go through. It’s something that you should avoid at all costs, if possible, but I also understand women have had to fight for this right, you know.
Interviewer: Yeah.
Participant: So, I think it is a right, that we are entitled to choose, because there’s all kinds of different circumstances as to why ladies end up getting pregnant. […] You know I think that we’re lucky in this country to be able to have that option.
Interviewer: Yeah. Okay
Participant: Very lucky. (SASS124, 24, England)

This extract highlights the complexities of disentangling negative and non-negative talk about abortion – which were so often enmeshed in the data – and a conflict between a theoretically positive orientation to abortion in the abstract, and an individual experience of it as nonetheless challenging. While this woman felt abortion was an ‘amazing option’, it nevertheless ‘should be avoided at all costs’ and is thus not straightforwardly positive. She was emphatic, however, that it is and should be something over which women have a right to choose and was one of the few participants in this sub-sample who framed abortion in terms of women’s rights. SASS124 was also among many participants who conveyed explicit awareness that this was not an option available to women universally.

SASS022 similarly framed her perspective in relation to the decision to seek abortion, and taking ownership thereof:

I was totally fine. We decided to go through with it, and I felt very calm and at peace with my decision. I didn’t feel like I was making the wrong decision – neither of us did. (SASS022, 22, Scotland)

Here and throughout, this woman’s account was strongly grounded in her relationship with her partner, and she noted that both ‘came to the decision together’. It appeared that the fact that she felt supported, and perceived seeking abortion to have been a joint decision, helped her to express this positively, although her presentation of it as something to feel ‘at peace with’ nevertheless implies the potential for judgement. She also contextualised her experience with that of a friend who had undergone an abortion not long before her:

She was the opposite from me. I was quite open with all my friends that I knew I could trust. I wasn’t just going out on the town and telling everybody, but she was very much kind of ashamed: ‘I didn’t really want to tell anybody’. She was like: ‘I know it’s the best decision I’ve ever made, ‘cause it was just a silly one-night-stand, but… it has kind of *affected* me, in a way.’ And I was, like, I felt really bad ‘cause I totally didn’t feel like that. I felt quite, totally just free about it (SASS022, 22, Scotland)

The contrast SASS022 explicitly sets up between her feelings and those of her friend foreground her own more positive approach. Noting that she ‘felt really bad’ for not feeling bad also once again highlights the pervasiveness of an underlying assumption that abortion is something about which one should feel negative.

### ‘I’m not ashamed’: negation statements

A second component of a normalising stock of knowledge identified in our analysis was a tendency for women to frame their views or experiences of abortion using negation statements, in a way which could be interpreted as not only using non-negative language, but actively challenging negativity. These were peppered throughout participants’ accounts, expressing what they did not think or feel about their experiences.

For many, negation statements related to negative emotions around the decisions and the procedure itself, explaining ‘I wasn’t devastated’ and ‘I wasn’t traumatised’, or ‘I don’t feel embarrassed’ and ‘I’m not ashamed’. Often this was linked to interaction with others, as was the case in the following account of interactions with health professionals:

…it was actually quite a good experience, I didn’t feel like, y’know, ashamed of what I was doing. Nobody made me feel kind of bad or guilty for what I was going through. (SASS062, 21, Scotland)

The option for SASS062 to not feel ashamed (or bad or guilty) was thus *supported by* the actions of those around her. Conversely, SASS007 explained how her actions (limiting who she told) were shaped in spite of her not feeling embarrassment:

I’m quite close to [work colleagues] as well, so I don’t, like, I wasn’t embarrassed by the situation, I would rather tell people than try to cover it up. […] just in terms of, [I] would rather less people knew than more, not ‘cause I’m embarrassed, but I just don’t want it to be something that’s kind of constantly brought up or made a big deal of… (SASS007, 19, Scotland)

A tension is evident here in the way this participant expressed not being embarrassed, in that it was in the context of still wanting to limit how many people knew about her (in this case two) abortions. The potential for judgement was also apparent in her account of her interactions with health professionals:

I didn’t feel kind of judged which I was a bit worried about, I didn’t want people to be like: ‘Oh,’ like: ‘She’s made the same mistake again’ kind of thing. Which was really nice, it wasn’t – I didn’t feel kind of patronised or like looked down upon. (SASS007, 19, Scotland)

The language choices in both these extracts are indicative of a potential for judgement that SASS007 keenly felt, and which SASS062 noted above that she was pleased to have avoided. In a similar vein, others highlighted that they were ‘not being stupid’ when they conceived and had used contraception but become pregnant anyway. All this language suggests a presupposition by women that the shared knowledge to which they were speaking is that abortion is a devastating, traumatic, embarrassing or shameful thing for a woman to undergo; and an assumption that they would be viewed as stupid or irresponsible for having conceived a pregnancy which they are unable or unwilling to continue. On the other hand, these negation statements can be viewed as women actively resisting or rejecting a default positioning of abortion in these ways.

In a particularly clear example of these negations SASS022 reflected on her experiences:

It wasn’t hindering me. It wasn’t, like, a weight on my shoulders that I was keeping secret or anything like that […] It wasn’t affecting me. It wasn’t making me upset. It wasn’t making me angry or sad or anything like that […] I don’t feel like I’ve had to ‘deal’ with something – it just happened… […] There hasn’t been a ‘what if’ and I don’t think there is gonna be. I don’t think I’m ever gonna regret them because, I mean, I haven’t so far and I’ve gone through it twice. (SASS022, 22, Scotland)

Negations also appeared in relation to what the literature terms the ‘prevalence paradox’, in which abortion is thought to be uncommon because it is little discussed (Kumar, Hessini, and Mitchell 2009). Some women noted, for example, that abortion is in fact ‘not uncommon and taboo’, the implication being that this was despite its typical framing. Regarding a perceived scarcity of experiential information on abortion which results from (and perpetuates) this misconception, SASS007 went on to say that, if advising a friend, she ‘…would stress that […] it’s not gonna affect you in the future. And […] as much as it’s not an enjoyable experience emotionally, it’s really not that bad physically’ (SASS007, 19, Scotland). This speaks to the major concern engendered in women by anti-abortion groups which spread misinformation on longer-term effects of abortion on physical and mental health (Rowlands 2011).

### ‘It was good – well, not good, but…’: constraints on non-negative talk

While much of the analysis presented so far alludes to constraints on non-negative talk around abortion, these were in some cases much more explicit and specific. Where this was so, these related primarily to a tendency to revise more explicitly positive framings, in particular in response to ‘real or imagined dialogue with others’.

There was a common tendency for participants to revise statements where they had been quite frank about aspects of their abortion experience, or to explicitly acknowledge that some might view their statement as problematic, perhaps for fear of sounding too glib. For some, this was in a general sense, as with SASS074 who said: ‘I’m glad-it sounds horrible - but I’m glad that I did it’. This qualification of her gladness suggests an acknowledgement of how she assumed the statement might be perceived, and that gladness – rather than, say, contrition – would be a potentially unacceptable feeling to express about abortion.

In other instances, such constraints appeared in relation to experiences of abortion services. SASS059, for example, explained:

…it was good – well, not ‘good’, but…the whole sort of experience was quite positive, and it was quite a comfortable sort of situation. A comfortable ‘environment’ is probably a better word for it. (SASS059, 21, Scotland)

SASS059’s revision of her initial language choice may suggest an unease at straying too far from what she perceived to be an acceptable narrative to present. Her choice of the word ‘comfortable’ is also interesting because of the way it appears to convey both her own feelings about the situation and her experiences of the environment, as well as the interaction between the two.

Much of what appeared in the data in relation to constraints on non-negative talk appeared to relate to the impact of women’s expectations of what the experience would be like, and/or how they should feel about it. In this sense, a further factor which appeared to shape non-negative talk, and what participants were comfortable saying in the context, was evident where participants described experiences of being in a clinic or hospital ward for treatment. Again, this was commonly couched in terms of an awareness of the experiences and reactions of those around them:

It wasn’t really a nice thing sitting in the [recovery] room with everyone. It really hits you, like, ‘Oh god, other people feel differently about it, they were really upset’. And there’s me like getting coffee and asking to leave straight away because I felt fine (SASS115, 23, England)

In terms of non-negativity, SASS115 noted here that she ‘felt fine’, but the fact that this did not appear to be congruent with the feelings of others around her made her uncomfortable. SASS120’s account echoed this:

I woke up and… they were like ‘how do you feel?’ and I was like ‘Fucking brilliant’. And like all these women were looking so sad and I was like ‘why did I say that?’ … [But] I literally felt great. (SASS120, 21, England)

Where these participants’ feelings or experiences seemed to them at odds with what they perceived of those around them, this appeared to result in their feeling guilty for not being upset, sad or repentant. Participants linked these feelings to their immediate surroundings, rather than directly to negative higher-level narratives around abortion. It does not seem a leap, however, to suggest that these feelings nonetheless indicate a constraint stemming from a broadly negative abortion narrative, in relation to which women were making meaning from their experiences.

## Discussion

Our analysis, grounded in the perspectives of women who have experienced abortion, shows what a normalising narrative might look like. It thus contributes to shifting debates around abortion away from a sole focus on stigma, while also highlighting how stigma continues to present a backdrop against which abortion narratives are constituted and perpetuated.

Overall, despite a dominant social narrative of abortion as negative, this is not the case for everyone. We identified examples across the secondary dataset in which abortion was experienced as a positive option or life event. Among other things, this speaks to Furedi’s (2016) notion of abortion not only as a legitimate option but as a moral choice, and the need for it to be positioned as such. Echoing Millar (2017), our analysis shows that abortion might be categorised for many as the ‘right choice’ and even, for some, a happy experience. This counters the position of abortion as fundamentally negative, and points instead to the adverse impact of a dominant sociocultural narrative in shaping what might otherwise be largely non-negative experiences.

However, the fact remains that explicit positivity in our data was rare. While narratives in some spheres may be shifting wholesale more toward the positive (Baird and Millar 2019), there is as yet limited evidence of this in the accounts of women in the UK who have undergone abortion. Moreover, where non-negative (including explicitly positive) language *did* appear, it was often intertwined with negative framings. This suggests not only the complexity of women’s feelings about abortion but also, echoing earlier findings, that ambivalence may be the most common response to abortion (Kero and Lalos 2000). We also observed a tension between framings of abortion in the abstract as a positive option which should be available, and abortion as a lived experience which is nevertheless challenging for individuals to undergo. This may be a point that those taking a ‘pro-choice’ stance are wary of making, but is one that is essential that we, as an academic and activist community, are comfortable to acknowledge (Hoggart 2015). Doing so is key to recognising the complex lived reality of women’s abortion experiences in this context (Purcell, Brown et al. 2017; Hoggart 2017).

As touched on earlier, we were struck by the absence of a sustained narrative of reproductive rights in women’s non-negative accounts of abortion, although this echoes Baird and Millar’s (2019) finding on the absence of ‘abortion politics’ from the web content they explored, and Hoggart (2017) finding that it was rare for women in her study to reject abortion stigma on grounds of its challenge to their bodily autonomy. Such absences are significant ‘given the weight accorded to rights and ‘choice’ discourses in abortion activism, specifically in Western contexts’ (Chiweshe, Mavuso, and Macleod 2017, 210). There is therefore a possible tension between the normalising narrative components we identify, and feminist-informed abortion activism, which has historically been grounded in an analysis of women’s reproductive control as empowering and essential to gender equality. The absence of a strong narrative of women’s rights can also be read as indicative of the context of our data. Despite lingering restrictions to access, abortion is perceived as widely available (in England, Scotland and Wales) and the fight for abortion rights as having been long won, rendering a rights-based narrative less immediately relevant. We have found this to be quite different in the Northern Irish data in our QSA study, on which we will report in future publications. In terms of action to be taken, what this also suggests is that a narrative grounded in women’s rights could (and may have to) be reworked, building on the sorts of non-negative language that we identify, in order to provide women with an expanded set of non-negative narrative resources on which to draw.

In narrative terms, and regarding the insight our methodological approach has afforded us, we wish to make three further points. First, one particularly interesting consideration relates to women’s use of negation in their accounts of abortion. As Norrick (2018) notes, negative statements are semantically weaker than positive ones, because they are less specific. That is, saying ‘I didn’t feel ashamed’ may suggest she felt the opposite of shame (for example pride or self-respect), but does not say that specifically, and leaves plenty of other options open. Whereas saying ‘I felt happy’ is significantly less equivocal, the more common use of less specific statements could be interpreted as indicative of women’s ambivalence about abortion.

A second significant consideration is that negation is typically used ‘as a denial of a proposition previously asserted, or subscribed to, or held as plausible by, or at least mentioned by, someone relevant in the discourse context.’ (Norrick 2018, 378, emphasis added). That is, by making negation statements, women alluded to negative judgements which they perceived to have been implied about their decision or experience. Given that this was not the case in any of the interviews, and that these terms were raised by interviewees themselves, this also points to the dominant – and predominantly negative – stock of knowledge or sociocultural narrative around abortion. This suggests another significant avenue of work toward a normalising narrative which removes the assumption of significant emotional difficulty and the default position that women seeking abortion should feel shame or contrition.

One further point regarding the use of negation is that it highlights a challenge faced by women if they wish to describe their experiences without using negative terms. The use of revisions in order to moderate positive statements suggest a concern with straying too far from a perceived dominant narrative, and of explicitly saying they feel positively about having undergone abortion. Even where they appear to have non-negative perspectives to share, they do not have readily available language to draw upon to do so, which limits them to explaining it in relation to those more well-rehearsed, dominant negative tropes. In other words, due to the limitations of culturally available narratives, women struggle to describe non-negative experiences.

### Limitations

A limitation to our analysis is that some of the data included is over ten years old. It thus does not reflect any recently emerging change that might be detected in newly produced data. We were also limited by the questions asked in the original projects, which did not probe for reflection on why women might have presented their accounts in the ways that they did. Broadly what we can say, however, is that our analysis highlights just how strongly embedded negativity and stigma are framings of abortion, making these difficult to challenge. Moreover, there is a clear indication of the need for further research to examine intersecting constraints on women’s ability to draw on non-negative framings of abortion; and the impact on their ability to do so of public attention to/interest in abortion, for example during decriminalisation or other campaigns.

## Conclusions

As a result of the approach taken in this paper, we suggest that a normalising narrative of abortion might incorporate: the default use of non-negative framings; an emphasis on contextualised positivity; and consideration of how abortion-related ‘negation statements’ might be translated into more semantically and socially powerful, positive language. The use of qualitative secondary analysis methodology, in particular, enabled exploration of those components across studies conducted in different times and places, and enhanced learning from those original datasets. Our approach was exploratory and small-scale and, as such, does not present an exhaustive picture. However, it is indicative of what such a narrative could consist of, were it to be given more credence and sociocultural prominence.

With regard to normalising abortion, our analysis highlights that abortion can be and is experienced non-negatively and, for some women, as a distinctly positive life event. This emphasises that there is nothing inherently ‘awful’ about abortion that determines that those undergoing it should be submerged in negative feelings. It also suggests that sociocultural narrative framings of abortion contribute significantly to this negativity, but that these in turn can be resisted and rejected. To revert to the words of one participant (SASS053), an overarching message regarding abortion is arguably that ‘Shit happens, you deal with it, you move on. It’s life’. With this in mind, the research, activist and provider communities should aim to support women to manage abortion in as pragmatic and unexceptional a way possible. We should also aim to explore ways to further normalise abortion in everyday life, by drawing on the power of claims to bodily autonomy, the politics of gender equality, and women’s lived experiences.

## Figures and Tables

**Table 1:** Summary of datasets.

Title	Jurisdiction	Sample	Data	Aim of original study
Project 1	Scotland	23 women who had sought abortion at 16+ weeks	23 interviews	To explore experiences of women in Scotland seeking abortion after 16 weeks of gestation.
Project 2	Scotland	46 women who had undergone early medical abortion	46 interviews	To explore women’s experiences of medical abortion under 9 weeks.
Project 3	Scotland	23 women who had undergone more than 1 abortion in 2 years	23 interviews	To explore experiences of women in Scotland who had sought more than one abortion in a two-year period.
Project 4	England	10 young women aged 16–19	10 interviews	To improve understanding of reasons behind, and explore opportunities to reduce, unintended and unwanted teenage pregnancy
Project 5	England and Wales	36 young women aged 16–24	51 interviews (two time points)	To understand influences on young women’s sexual behaviour before and after abortion.

## Footnotes

1
**Note**
Although partially legalised over 50 years ago in Scotland, England and Wales, abortion remains a criminal offence with certain exceptions, carrying a maximum sentence of life imprisonment. The campaign for full decriminalisation is supported by a broad range of bodies including the Royal Colleges of Obstetricians and Gynaecologists, General Practitioners and Midwives.
